# Supplementary figures and images for: Flavonoid Synthesis-Related Genes Determine the Color of Flower Petals in Brassica napus L
Source: Int J Mol Sci. 2023 Mar 30;24(7):6472. doi: 10.3390/ijms24076472 (PMC10094890; doi:10.3390/ijms24076472)

## Supplementary Figure S1.

(A) (Positive ion mode)

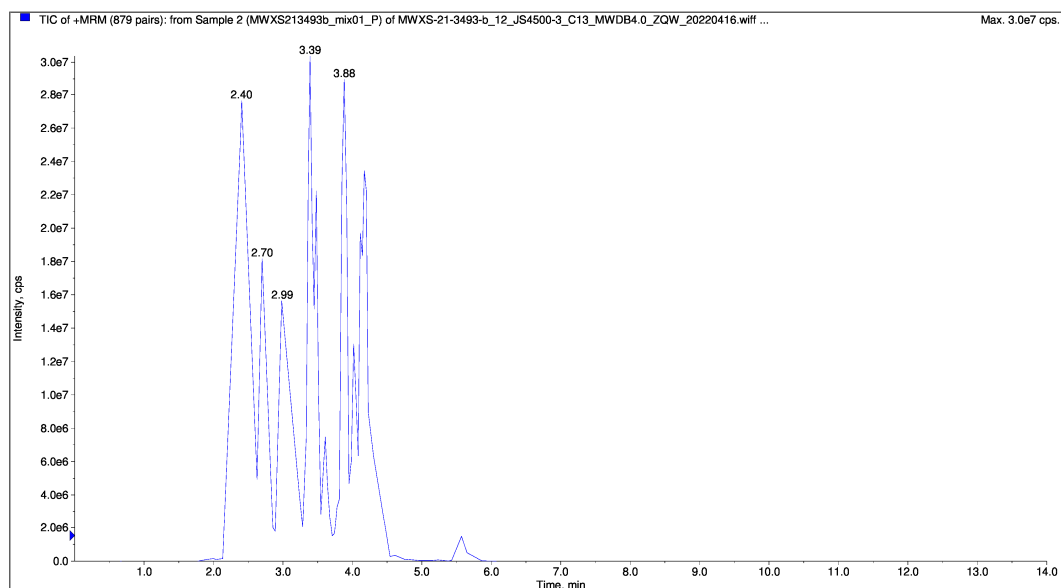

(B) (Positive ion mode)

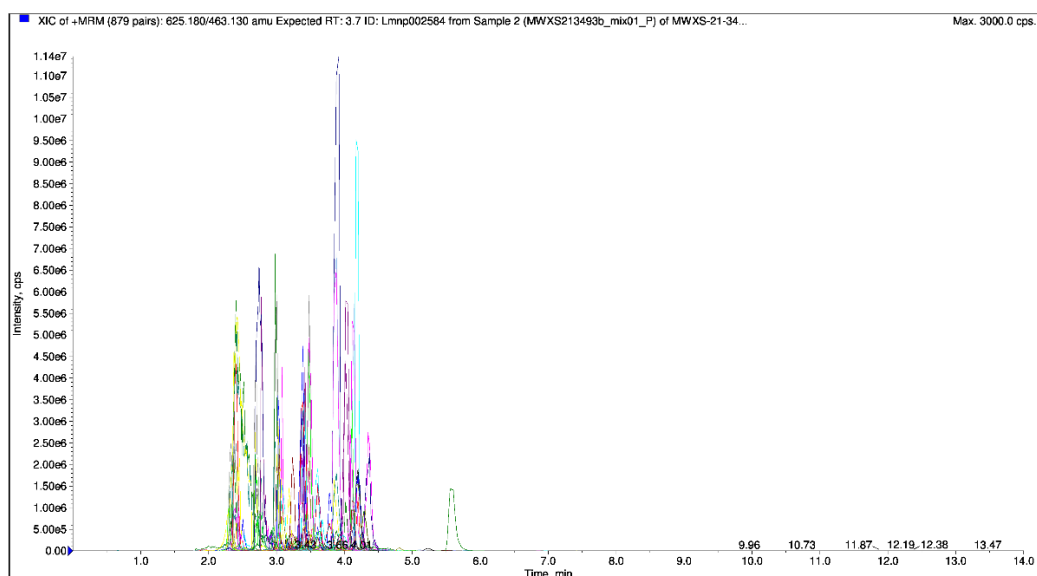

(C)

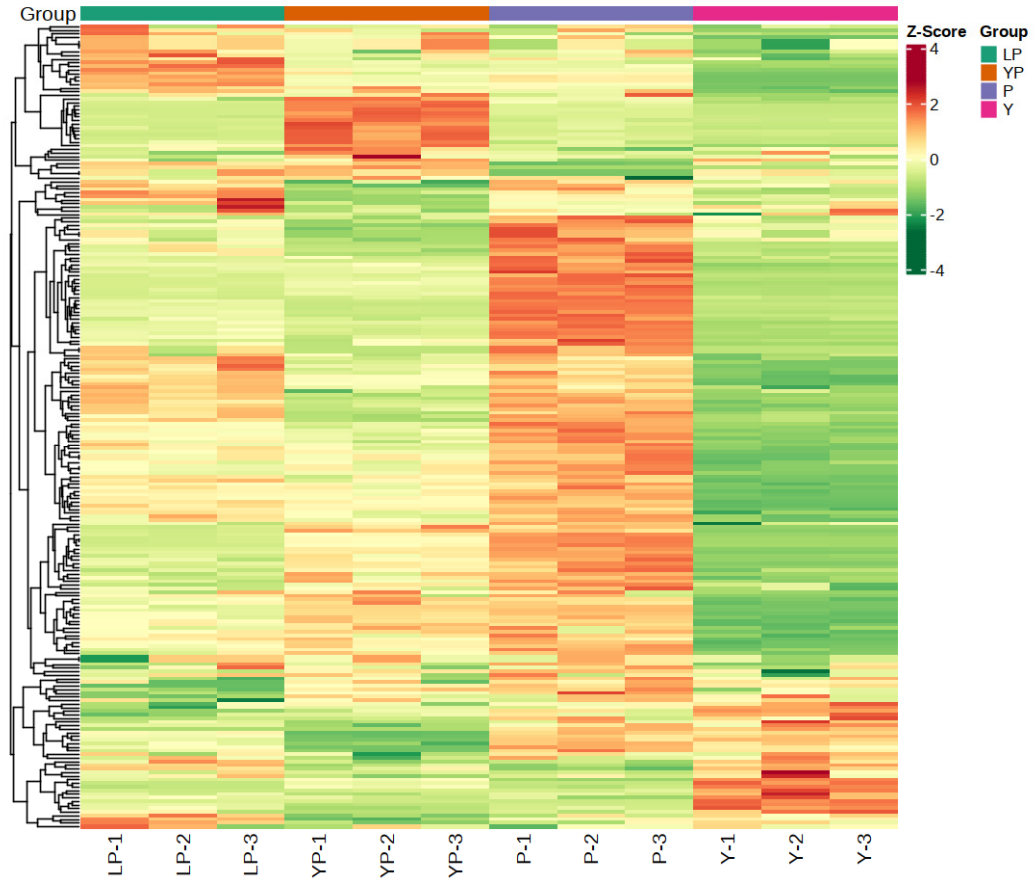

Supplement: Supplementary file 1 [file ijms-24-06472-s001.zip › Supplementary Figure S1.pdf]

### Supplementary Figure S2.

(From left to right: Y vs LP, Y vs YP, and Y vs P)

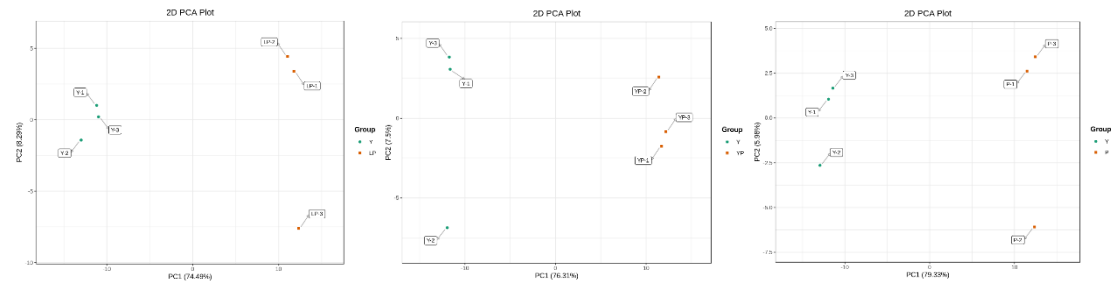

Supplement: Supplementary file 1 [file ijms-24-06472-s001.zip › Supplementary Figure S2.pdf]

Supplementary Figure S3.

(A) (From left to right: Y vs LP, Y vs YP, and Y vs P)

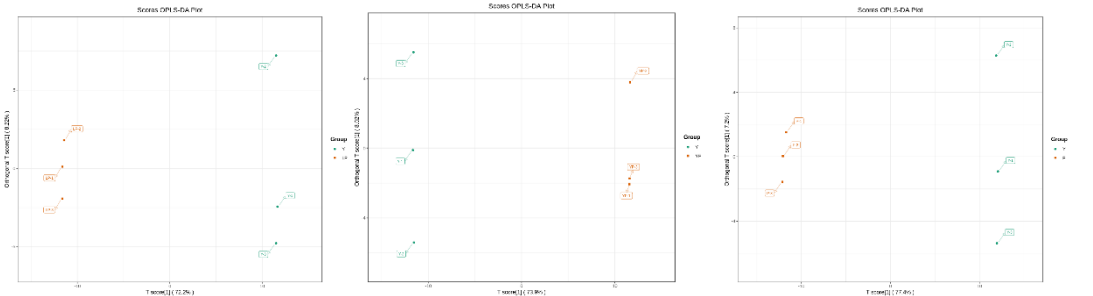

(B)

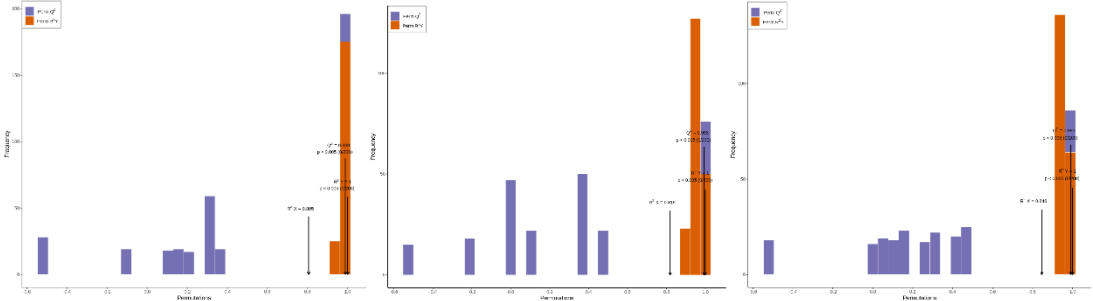

(C)

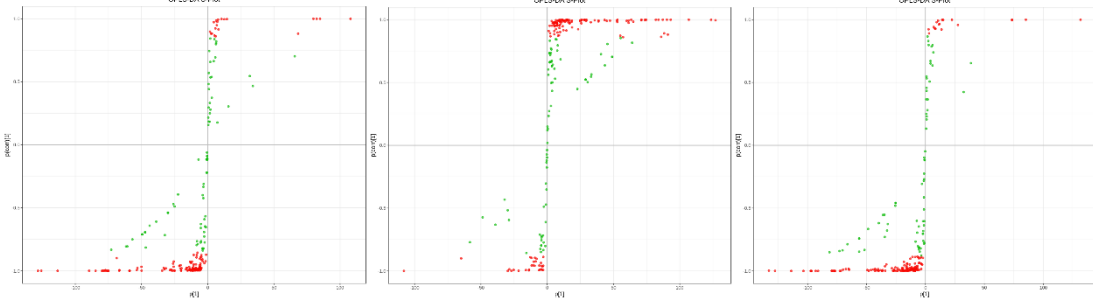

(D)

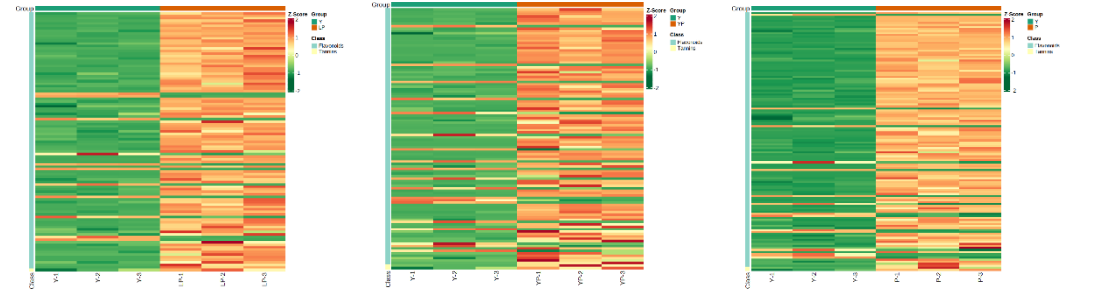

Supplement: Supplementary file 1 [file ijms-24-06472-s001.zip › Supplementary Figure S3.pdf]

Supplementary Figure S4.

(A)

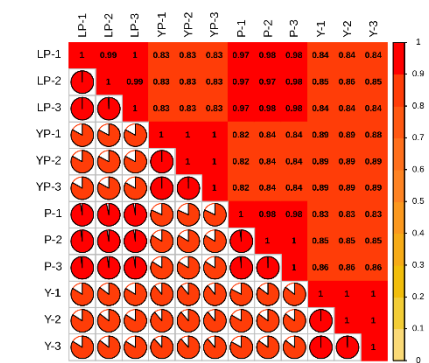

(B)

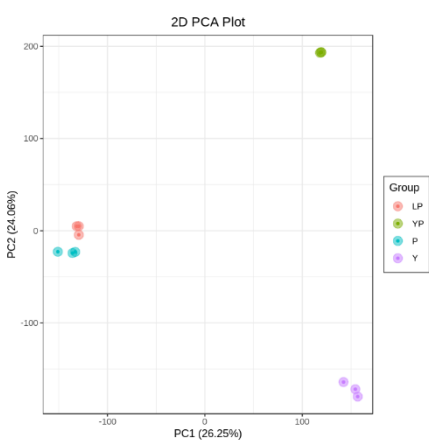

Supplement: Supplementary file 1 [file ijms-24-06472-s001.zip › Supplementary Figure S4.pdf]
